# An estimation of osteochondrodysplasia prevalence in Australian Scottish Fold cats: a retrospective study using VetCompass Data

**DOI:** 10.1186/s12917-023-03811-0

**Published:** 2023-11-29

**Authors:** Brandon D. Velie, Tracey Milden, Hannah Miller, Bianca Haase

**Affiliations:** 1 School of Life and Environmental Sciences, Faculty of Science, The University of Sydney, Camperdown, 2006 NSW Australia; 2 Sydney School of Veterinary Science, Faculty of Science, The University of Sydney, Camperdown, 2006 NSW Australia

**Keywords:** Feline, Skeleton, Arthritis, Cartilage

## Abstract

**Background:**

All Scottish Fold cats are believed to be affected by osteochondrodysplasia, a painful degenerative joint disorder. This retrospective study aimed to estimate the prevalence of osteochondrodysplasia in Scottish Fold and Scottish Straight cats in Australian veterinary clinics using electronic patient records (EPRs), collected between 1992 and 2018.

**Results:**

Consultation events (34,926) in EPRs from veterinary clinics located in New South Wales, Queensland, and Victoria, were collected from 1,131 Scottish Fold and 117 Scottish Shorthair cats. A clinical diagnosis of osteochondrodysplasia was made in 12/1,131 Scottish Fold cats. Additionally, 69 cats were identified with suspected osteochondrodysplasia. Of these, 64 were Scottish Fold and 5 were Scottish Shorthair cats. Male and female cats were equally represented. However, a significant difference was observed for the age clinical signs were first recorded in the EPRs. Cats diagnosed clinically with osteochondrodysplasia were significantly younger (*p* < *0.0001*) compared to cats identified as suspected SFOCD cases.

**Conclusions:**

Findings from this study suggest a relatively low prevalence of clinically diagnosed Scottish Fold osteochondrodysplasia (SFOCD) in the studied Australian Scottish Fold population, with cats generally diagnosed with SFOCD at less than 30 months of age. Further evidence is required to accurately assess the clinical relevance of SFOCD in the Scottish Fold population.

## Background

Scottish Fold cats are characterised by their forward-folded ears, an autosomal dominant trait known to affect cartilage development and maturation [1–3]. While the breed-defining ear folding in Scottish Fold cats appears benign, a progressive degenerative osteochondrodysplasia has been associated with the forward folded ear phenotype [4]. Early studies indicated that this progressive syndrome only affected cats homozygous for the underlying fold mutation; however, recent evidence suggests that any cat carrying at least one copy of the variant associated with ear-folding can develop osteochondrodysplasia [1, 2, 4–7]. Scottish Fold osteochondrodysplasia (SFOCD) is defined by skeletal abnormalities including narrowed joint spaces and irregularly shaped bones of the lower extremities, and while associated clinical signs such as lameness, stiff and inflexible tail can be indicators for the presence of the disorder, the diagnosis of SFOCD requires radiographic evidence of the previously described skeletal abnormalities [1, 7].

The variant associated with ear-folding in Scottish Fold cats is located in the *transient receptor potential vanilloid-type 4* (*TRPV4)* gene, a gene expressed in a range of tissues and immune cells [8–18]. The gene encodes for a gated ion channel pivotal for a range of physiological processes and its correct activation is crucial for the normal differentiation of chondrocytes, osteoblasts and osteoclasts [8, 18, 19]. While clinical and radiographic assessments of cats homozygous and heterozygous for the *TRPV4* variant support an association with osteochondrodysplasia, they have also provided evidence of high variability, in regard to severity as well as age of onset of clinical signs [2, 7]. Unfortunately, despite the well-recognised status of the disorder, previous studies have focused on severe clinical cases [1, 2, 4–6]. As a result, little is known about the prevalence of clinical SFOCD in the global Scottish Fold cat population beyond that of severe cases. Considering the recognised status of Scottish Fold osteochondrodysplasia as a painful, progressive syndrome and the increasing trend of banning breeds, studies investigating the prevalence and severity of clinically diagnosed and clinically suspected SFOCD cases across a range of countries/populations are needed. Therefore, the current study investigates the prevalence of SFOCD in Australian Scottish Fold cats using data from VetCompass Australia [20].

## Methods

Electronic patient records (EPRs) for Scottish Fold and Scottish Shorthair cats collected between 1992 and 2018 were provided by VetCompass Australia (https://www.vetcompass.com.au/). As SFOCD has been associated with ear folding, Scottish Shorthair cats were included as these cats are the offspring of Scottish Fold mating’s that lack the breed-defining ear folds (https://nzcf.com/breed/breeders/Scottish_Shorthair). All analyses were performed using the statistical software R version 4.0.2 [21].

### Prevalence estimation of clinically diagnosed SFOCD

All EPRs were searched for the presence of key words indicating the clinical presence of SFOCD. Search terms used were osteochondrodysplasia and OCD. EPRs containing at least one of the key words were then manually reviewed (i.e., individually assessed by the authors) to confirm “diagnosed” SFOCD case status. As a diagnosis of SFOCD requires a radiographic assessment, only cats reported in their EPRs as affected by SFOCD based on a radiographic assessment were considered as ‘diagnosed clinically’ with SFOCD (e.g., “xrays show mild changes consistent with SFOCD”).

### Prevalence estimation of clinically suspected SFOCD

Based on commonly reported clinical signs and radiographic findings of SFOCD in previous literature, all EPRs were searched for the presence of clinical key words that are likely to be associated with the presence of SFOCD [1, 2, 4, 5, 22, 23]. These clinical key words used were: osteochondrodysplasia, OCD, degenerative joint disease, DJD, osteoarthritis, OA, ankylosing, ankylosis, spondylosis, osteosclerosis, osteophyte, enthesophyte, osteochondroma, lameness, lame, limping, reluctance to jump, stiff, stilted gait, joint pain, reluctance to move, short limb, swelling, narrow joint space, inflexible tail, short tail, osteopenia, exostosis. All EPRs containing at least one of the search key words were manually reviewed. When EPRs contained at least one of the key words but information about the aetiology of the disorder was not clear or not provided, the cat was classified as a ‘clinically suspected’ case. To assess the impact of the ear phenotype, cats identified as clinically suspected SFOCD were separated by recorded ear phenotype (i.e., Scottish Fold vs Scottish Shorthair).

### Age, breed, and sex distribution between diagnosed clinically and clinically suspected SFOCD cats

To assess the age distribution amongst cats diagnosed clinically and clinically suspected SFOCD, age was calculated in months for each cat based on its earliest EPR that contained at least one of the aforementioned key words. A Shapiro–Wilk was used to test for normality and differences in age of first recording were subsequently investigated using a two-tailed *t*-test assuming unequal variances. To assess if ear phenotype influenced age at first recording, cats within each group (“diagnosed clinically” or “clinically suspected”) were separated based on their recorded phenotype and the analysis repeated.

Differences between sexes and breeds were calculated using a Chi-Square test for Independence. *P*-values of < 0.05 were considered significant. Additional demographic factors such as weight, housing, neutering status were unable to be assessed as the information was not included in the EPRs.

## Results

**Fig. 1:**
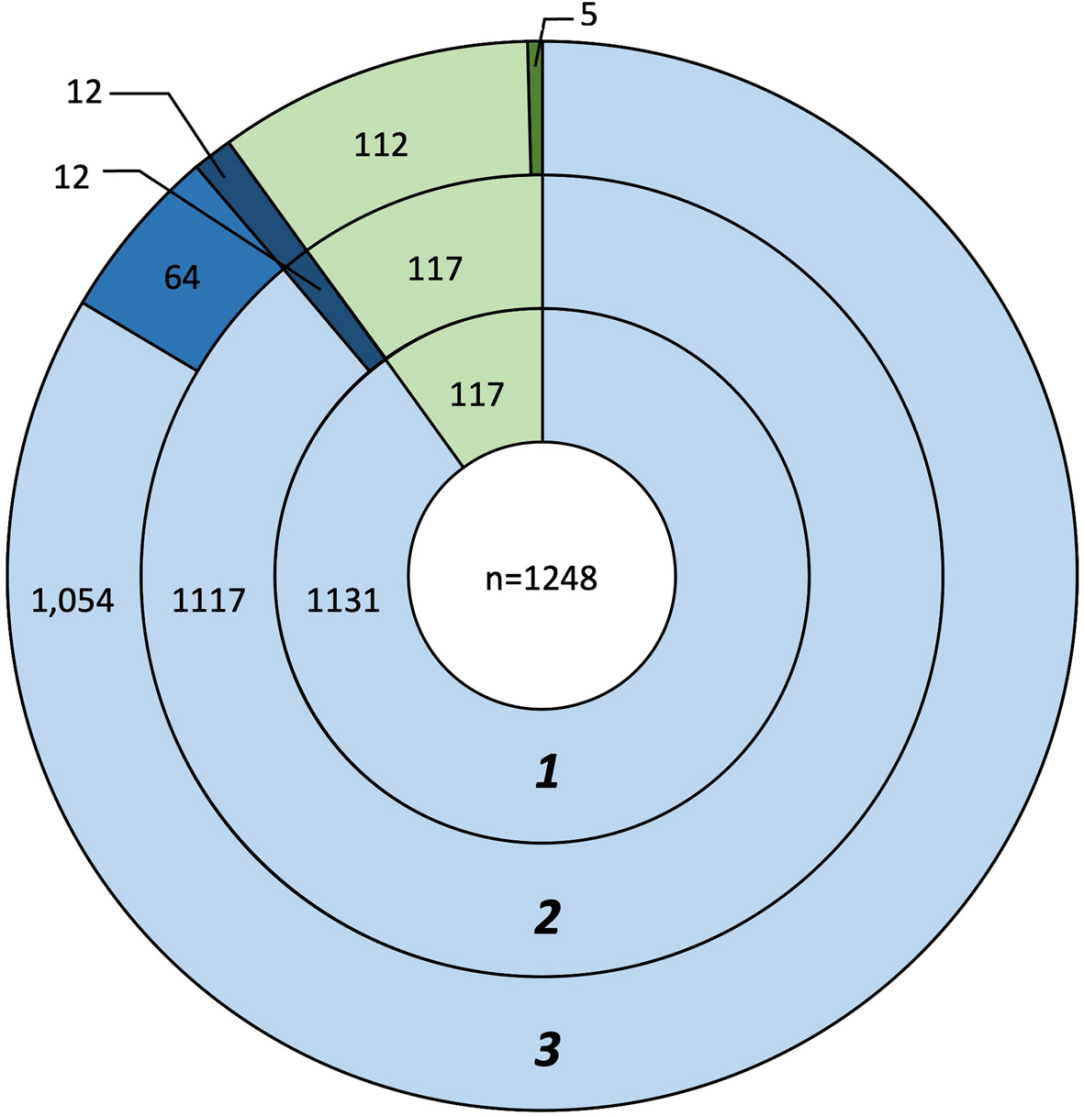
Distribution of cats identified as affected or possibly affected by Scottish Fold Osteochondrodysplasia. The total number of individuals included in the analysis is indicated in the centre. Each ring represents the outcome of an analysis with (1) representing the numbers of Scottish Fold cats (blue) and Scottish Shorthair cats (green) included in this study; (2) representing results of the first key word search for SFOCD affected cats. Cats identified as diagnosed with SFOCD are indicated in a dark shade of the phenotype defining colour; (3) representing results of the second key word search for possibly SFOCD affected cats. Cats identified as clinically suspected SFOCD in this analysis are indicated in a second shade of the breed defining colour

All Electronic Patient Records (EPRs) for Scottish Fold and Scottish Shorthair cats were provided by VetCompass Australia, which harvested EPRs from 139 veterinary clinics located in New South Wales, Queensland, and Victoria, Australia during the study period. A total of 34,926 individual patient records were retrieved from 1,248 cats, of which 1,131 were reported as Scottish Fold and 117 as Scottish Shorthair cats (Fig. 1-1).

### Prevalence estimation of diagnosed clinically SFOCD cats

**Fig. 2:**
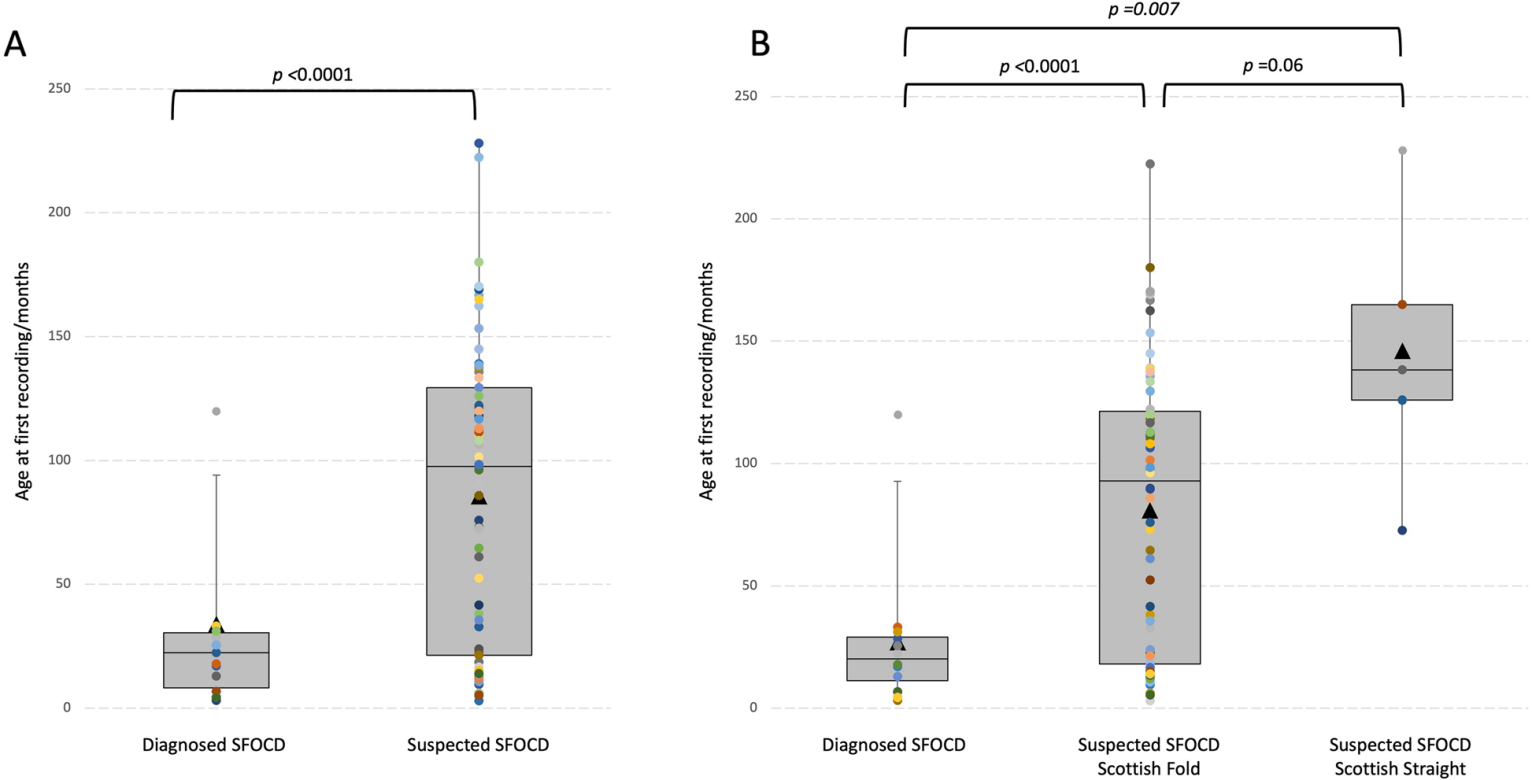
Age clinically diagnosed SFOCD and clinically suspected SFOCD cats. Age (months) of cats identified as, (**A**) diagnosed clinically SFOCD and clinically suspected SFOCD; and (**B**) as diagnosed clinically SFOCD and clinically suspected SFOCD, separated based on recorded ear phenotype; Boxes indicated the 25^th^ and 75^th^ percentiles, the horizontal line denotes the median, and the triangle within the box plot indicates the mean; individuals are indicated as dots

The EPRs for 18/1,248 cats were identified as having key words in their EPRs indicating the clinical presence of SFOCD. Manual review of individual EPRs resulted in the exclusion of six cats (e.g., "…no abnormalities were detected with the X-ray.”), thus 12/1,248 cats were classified as diagnosed clinically with SFOCD, all of which were Scottish Fold (Fig. 1-2).

### Prevalence estimation of clinically suspected SFOCD cats

A total of 208/1,248 cats had EPRs retrieved with words indicating the clinical presents or are suggestive of SFOCD. Of these, 187 were listed as Scottish Fold cats and 21 as Scottish Shorthair cats. All 18 cats identified in the previous search were also identified in this second search using the extended set of keywords. The manual review of each cat’s EPRs resulted in the exclusion of 111/187 Scottish Fold cats and 16/21 Scottish Shorthair cats as no evidence of SFOCD could be found in the records (e.g. “…ingrown nails…”). The remaining 76 Scottish Fold cats included the 12 Scottish Fold cats previously confirmed as diagnosed clinically with SFOCD. This resulted in a total of 64 Scottish Fold and 5 Scottish Shorthair cats with clinically suspected SFOCD (Fig. 1-3).

### Age and sex distribution

Seven males and five females were diagnosed clinically with SFOCD and an additional 37 males and 32 females were identified as clinically suspected SFOCD. There was no significant sex difference between the groups (*p* = *0.76*).

In the assessed dataset, the age of first recording was not normally distributed between groups (diagnosed clinically vs. clinically suspected). Cats diagnosed clinically with SFOCD were significantly younger (*p* < 0.0001) compared to cats clinically suspected of having SFOCD (Fig. 2A). Cats diagnosed clinically with SFOCD had a median age of 20 months compared to a median age of 98 months for clinically suspected SFOCD cats. One cat was diagnosed with SFOCD at the age of 119 months (9.9 years), the remaining cats’ EPRs place clinical diagnosis with SFOCD between 3 and 29 months of age (median 20 months, IQR 22 months). In contrast, the age of cats with clinically suspected SFOCD ranged from 3 to 228 months (19.0 years) of age (median 98 months, IQR 108 months). However, the first EPRs for 30% (20/69) for clinically suspected SFOCD cats occurred at less than 29 months of age.

As all cats identified as diagnosed clinically with SFOCD were recorded as a Scottish Fold an analysis based on ear phenotype was not possible. When clinically suspected SFOCD cats were separated by recorded ear phenotype (Scottish Fold vs Scottish Shorthair), age at first recording of SFOCD associated keywords ranged from 3 to 222.3 months (median 93 months, IQR 103 months) for Scottish Fold cats (*n* = 64), while it ranged between 72.6 and 228 months for the Scottish Shorthair cats (*n* = 5) (median 138 months, IQR 39 months) (*p* = *0.06*) (Fig. 2B). A significant difference (*p* = *0.007*) between age at first EPR entry was observed between cats diagnosed clinically with SFOCD and Scottish Shorthair cats clinically suspected as SFOCD affected.

## Discussion

Scottish Fold cats identified in this study as diagnosed clinically with SFOCD were generally less than 30 months of age and diagnosis in all 12 Scottish Fold cats was supported by patient records either directly referring to radiographic findings or that a diagnosis was made by a previous veterinarian. Scottish Fold osteochondrodysplasia (SFOCD) refers to skeletal changes and these skeletal abnormalities can be accompanied by a range of clinical signs, especially in severe cases [7]. Clinical signs of SFOCD are similar to those observed in cats affected by osteoarthritis [24–27]. While osteoarthritis is common in cats, skeletal changes are most commonly seen in cats six years and older [25, 26, 28]. Due to the young age at diagnosis, it is unlikely these cats were misclassified and that the skeletal changes observed were due to age-related osteoarthritis [2, 6, 22, 24, 29].

Overall, EPRs indicated a low prevalence of clinically diagnosed (1.1%) and clinically suspected SFOCD (5.7%) within this sample of Scottish Fold and Scottish Shorthair cats. It is possible that cases, or potential cases, were missed given that the details within EPRs are variable, and clinical information provided may have been too general for the search parameters, contained severe typographical errors, or information was not entered. Furthermore, records of individual cats may be incomplete or not available as owners may use several veterinary clinics over the lifetime of an animal and not all practices contribute their data to the VetCompass project. This may have resulted in an underrepresentation of Scottish Fold cats classified as diagnosed clinically or clinically suspected of having SFOCD. Scottish Shorthair cats are offspring of Scottish Fold matings that did not inherit the *TRPV4* variant and based on current evidence, SFOCD is associated with this variant [4]. Furthermore, SFOCD has yet to be described in Scottish Shorthair cats [2]. The Scottish Shorthair cats identified as clinically suspected to have SFOCD had an average age of 145 months. While the EPRs did not allow for an assessment of the underlying aetiology, the key words identified are likely the result of normal age-related osteoarthritic changes rather than SFOCD in these cats. Osteoarthritis is common in cats and while the reported radiographic prevalence varies between studies, current evidence suggests that most cats six years and older have osteoarthritic changes in at least one joint [24, 28, 30]. Interestingly, the median age at which Scottish Fold cats were identified as suspected SFOCD cases was 93 months, with 20 cats being 28 months or younger and 36 cats being older than 72 months (6 years). As radiographs are not available in VetCompass, we were unable to validate if the observed clinical signs were due to normal age-related changes or caused by SFOCD. In the suspected SFOCD group, the aetiology in cats older than 72 months is unclear (*n* = 36), the age of the other 20 animals matches the age of animals clinically diagnosed with SFOCD in other studies [1, 5, 6]. While it was not possible to establish the exact diagnosis, based on current evidence it is likely that at least some of these animals are affected by SFOCD [1, 5]. Utilising key word searches are useful as they enable the extraction of potentially relevant cases from large datasets. However, manual assessment of extracted records was pivotal as it allowed for the assessment of the clinical context in which these key words were used and enabled the exclusion of false positives.

The majority of previous studies have focused on Scottish Fold cats severely affected by SFOCD [1, 4–6] and while current findings suggest that all Scottish Fold cats are affected by SFOCD to some extent, the overrepresentation of severe cases in current literature has likely resulted a breed bias. A blinded assessment of radiographs of heterozygous Scottish Fold cats and Scottish Shorthair cats by multiple experienced veterinary radiologists demonstrated a trend towards more extensive changes in Scottish Fold cats; however, the severity of skeletal abnormalities was less then reported in previous studies and none of the Scottish Fold cats assessed showed clinical signs associated with SFOCD [1, 2, 4, 6, 7]. This further supports findings that assessing skeletal changes and their clinical relevance is challenging [26].

Overall, findings from this study suggest a relatively low prevalence of Scottish Fold cats diagnosed clinically with SFOCD, with affected cats generally being less then 30 months of age. Scottish Fold cats identified as clinically suspected of having SFOCD showed a large age difference for first recorded onset of clinical signs. Further research is required to accurately assess the extent of SFOCD and its clinical implications. In addition, further research into SFOCD, its risk factors, and prevalence in the general Scottish Fold population are necessary to better understand the aetiology of this disorder and enable more informed discussions.

## Conclusion

Results from this study indicate a relatively low prevalence of Scottish Fold cats diagnosed clinically with SFOCD (1.1%; 12/1,131 Scottish Fold cats), and that the diagnosis of SFOCD is generally made at 30 months of age or younger. Our findings together with findings from other studies indicated that closely monitoring the skeletal health of Scottish Fold cats and excluding animals with early signs of SFOCD (before 30 months) from breeding programs could potentially improve the overall health of Scottish Fold cats.

## Data Availability

Data may be obtained by a third party and are not publicly available. Data are available on agreement with VetCompass Australia ( info@vetcompass.com.au).
